# Functional Foods and Nutraceuticals to Reduce the Risk of Cardiometabolic Disease: Where We Are, and Where We Are Going

**DOI:** 10.3390/nu16183152

**Published:** 2024-09-18

**Authors:** Federica Fogacci, Claudio Borghi, Arrigo Francesco Giuseppe Cicero

**Affiliations:** 1 Hypertension and Cardiovascular Risk Research Unit, Medical and Surgical Sciences Department, Alma Mater Studiorum University of Bologna, 40138 Bologna, Italy; claudio.borghi@unibo.it; 2 Italian Nutraceutical Society (SINut), 40138 Bologna, Italy; 3 Cardiovascular Medicine Unit, IRCCS Azienda Ospedaliero-Universitaria di Bologna, 40138 Bologna, Italy

Atherosclerotic cardiovascular diseases (ASCVDs) remain leading causes of mortality and disability in Western countries. Primary prevention is recognized as essential for decreasing the incidence of ASCVDs and mitigating the economic burden on both individuals and the healthcare system [1,2]. “Interventional medicine” prioritizes lifestyle modifications as the initial therapeutic approach, emphasizing a healthy diet and regular physical activity [1,3]. In fact, 13 food-related risks explain a large part of ASCVD incidences and related mortality in Western countries [4]. Additionally, extensive research has identified various food and plant bioactives with potential efficacy in preventing and mitigating the most prevalent cardiovascular risk factors such as hypercholesterolemia, hypertension, vascular inflammation, and impaired vascular compliance [5,6]. Certain lipid- and blood-pressure-lowering bioactives have been investigated for their beneficial effects on vascular health, particularly endothelial function and arterial stiffness [7,8]. Numerous nutraceuticals have demonstrated additive or synergistic effects when used in combination, which sometimes allows for reduced dosages of extracts and achieves a “multi-factorial” impact on multiple cardiovascular risk factors [9,10].

In this context, the articles published in the current Special Issue are of particular interest. The Mediterranean diet has demonstrated preventive efficacy, increasing the plasma level of nitric oxide and prostacyclin and decreasing thromboxane B_2_, even in patients affected by Prinzmetal angina [11]. These data support previous data showing how a high adherence to the Mediterranean diet is associated with an improvement in endothelial function [12] and atherosclerosis progression [13]. In fact, the positive effects of the Mediterranean diet can be enhanced with the use of specific dietary supplements containing nutrients and/or bioactive compounds that offer health-promoting properties [14,15]. For instance, dietary supplementation with polyphenols can exert a significant effect on plasma lipids and hepatocyte metabolism [16]. This could be particularly interesting considering that non-alcoholic fatty liver disease prevalence is about 30% all over the world and currently, we do not have effective and safe drug treatments available [17]. The addition of different types of microalgae to the diet can also result in different metabolic effects [18]. For instance, the intake of 15 g of *Chlorella* (dry weight) for 14 days was associated with a significant improvement in plasma lipid and decreased selenium, iron and ferritin. Dietary supplementation with 15 g of *Microchloropsis* (dry weight) resulted in increased omega-3 fatty acids plasma levels, and in decreased levels of vitamin D. However, both these algae were associated with increased levels of uric acid in serum and urine. Therefore, the balance between the positive and negative effects of microalgae in human nutrition must be carefully evaluated based on individual clinical conditions [19].

One of the most investigated dietary supplements is red yeast rice, with a well-recognized lipid-lowering effect and a safety profile that seems conditioned more by the industrial process quality associated with its production than the product itself [20,21].

Even negative data are useful when they provide potentially relevant information. In this Special Issue, Ehret et al. tested the 12-week effect of fiber-enriched foods on the feeling of satiety and self-perceived quality of life, without finding any difference between the actively treated individuals and those taking a placebo [22]. In addition, Uffelman et al. carried out a systematic review showing that mushroom consumption is associated with a mild but clinically irrelevant improvement in triglycerides and high-sensitivity *C*-reactive protein levels, without any effect on glucose metabolism or blood pressure [23]. This does not mean that dietary fibers do not have a positive impact on satiety [24] or that mushroom intake is not associated with an improvement in cardiovascular risk factors [25], but that more research is needed to understand which fibers and which mushrooms could exert the most positive impact on human health.

In conclusion, further epidemiological and clinical research is needed to understand which dietary components and bioactive compounds should be added to an overall healthy diet to further prevent ASCVD and increase longevity [26,27].
